# ﻿Discovery of five new species of *Allacta* from Yunnan and Hainan, China (Blattodea, Pseudophyllodromiidae)

**DOI:** 10.3897/zookeys.1191.113043

**Published:** 2024-02-05

**Authors:** Bian-Lun Li, Peng-Hui Hu, Lin Guo, Yan-Li Che, Zong-Qing Wang

**Affiliations:** 1 College of Plant Protection, Southwest University, Beibei, Chongqing 400715, China Southwest University Chongqing China; 2 Key Laboratory of Agricultural Biosafety and Green Production of Upper Yangtze River (Ministry of Education), Southwest University, Chongqing 400715, China Southwest University Chongqing China

**Keywords:** ABGD, checklist, cockroach, cryptic species, DNA barcodes, female genitalia, identification key

## Abstract

We examined new *Allacta* materials from Yunnan and Hainan Province, China, and discovered new species using both morphological and molecular species delimitation (ABGD) methods. Five new species are described: *A.bifolium* Li & Wang, **sp. nov.**, *A.hemiptera* Li & Wang, **sp. nov.**, *A.lunulara* Li & Wang, **sp. nov.**, *A.redacta* Li & Wang, **sp. nov.**, and *A.unicaudata* Li & Wang, **sp. nov.** All five species are placed under the *hamifera* species group. An updated key and checklist of *Allacta* species from China are provided.

## ﻿Introduction

The genus *Allacta* Saussure & Zehntner, 1895 belongs to Pseudophyllodromiidae, with 47 known species mainly distributed in the Oriental and Australasian regions (Beccaloni 2014; He et al. 2019; Prabakaran et al. 2019; Senraj et al. 2021). Species of this genus are found inhabiting tree trunk surfaces at night or under the barks in the daytime (He et al. 2019). They are distinguished from other Pseudophyllodromiidae by the tarsal pulvillus only present on the fourth tarsomere. Recently, Prabakaran et al. (2019) and Senraj et al. (2021) described four new *Allacta* species from India, all with a solidly dark pronotal disk, broadly V-shaped or keel-like male interstylar margin, and all belonging to the *hamifera* species group (Roth 1993b).

Eight species were recorded from China according to He et al. (2019). However, the specimen depository of *Allactahainanensis* (Liu et al. 2017) was not assigned (see Liu et al. 2017). Therefore, this species is invalid according to the International Code of Zoological Nomenclature (see Article 16.4.2) (ICZN 1999).

DNA barcoding has been widely used in cockroach identification in recent years, but is considered more reliable when used in combination with morphological evidence (Evangelista et al. 2013; Che et al. 2017; Yang et al. 2019; He et al. 2021). Although male genital morphology is traditionally used in cockroaches, female genitalia characters have also been shown to be useful in the identification of cockroaches, such as genera *Cryptocercus* and *Anaplecta* (Bai et al. 2018; Zhu et al. 2022).

In this study, newly collected *Allacta* materials from Yunnan and Hainan Provinces were examined, and they were found morphologically different from the known species. Based on morphological characters as well as the ABGD approach, five new species were confirmed, including the establishment of *Allactahemiptera* Li & Wang, sp. nov. for the *nomen nudum Temnopteryxhainanensis*Liu et al., 2017.

## ﻿Materials and methods

### ﻿Morphological examination

Twenty-six studied specimens of *Allacta* were collected from Yunnan, Hainan and Xizang Provinces and were deposited in College of Plant Protection, Southwest University, Chongqing, China (**SWU**) and Shanghai Entomology Museum, Shanghai, China (**SEM**). Morphological terminology used in this paper follows Roth (2003), McKittrick (1964) and Li et al. (2018). Vein abbreviations in the figures are as follows:

**CuA** cubitus anterior;

**CuP** cubitus posterior;

**M** media;

**R** radius;

**RA** radius anterior;

**RP** radius posterior;

**ScP** subcosta posterio;

**V** vannal;

**Pcu** postcubitus.

All materials are preserved in absolute ethanol and stored at -20 °C. The genitalia were handled based on a standard procedure in which terminal segments of the abdomen were cut off, heated in tap-water and rinsed with sterile water to remove trace amounts of NaOH, and then immersed in glycerol for further photography, dissection and preservation. Photos were taken with Leica DFC camera through a Leica M205A stereomicroscope; dissection and observation were performed under a Motic K400 stereomicroscope. All photos and images were edited with Adobe Photoshop CC 2019.

### ﻿DNA sequencing

Total DNA was extracted from hindleg tissues by the Hipure Tissue DNA Mini Kit (Magen Biotech, Guangzhou), and the remaining body parts were stored in absolute ethanol as voucher specimens. Primers for PCR amplification were LCO1490 (5’-GGTCAACAAATCATAAGATATTGG-3’) and HCO2198 (5’-TAAACTTCAGGGTGACCAAAAAATCA-3’) (Folmer et al. 1994). The PCR reactions were carried out in a 25 μL volume. The amplification conditions were: initial denaturation at 98 °C for 2 min, followed by 35 cycles for 15 s at 98 °C, 10 s at 49 °C, and 1 min at 72 °C, with a final extension of 5 min at 72 °C. All DNA purification and sequencing were carried out by Tsingke Biotech Co., Ltd. (Beijing, China) using the aforementioned primers.

### ﻿Sequence processing and phylogenetic analyses

A total of 30 *COI* sequences were analyzed, including 18 newly obtained sequences from this study; eight sequences representing eight *Allacta* species downloaded from GenBank; and four sequences represent the outgroup from four genera (*Margattea* Shelford, 1911, *Sorineuchora* Caudell, 1927, *Balta* Tepper, 1893 and *Shelfordina* Hebard, 1929) of Pseudophyllodromiidae (Table 1). Sequences were assembled and aligned using Geneious Prime 2023.1.2 (Kearse et al. 2012) and MEGA 7.0 (Kumar et al. 2007), respectively. Intraspecific and interspecific genetic divergences were computed using MEGA 7.0 based on the Kimura 2-parameter (K2P) distance model (Kimura 1980). A Maximum likelihood (ML) tree was constructed in PhyloSuite v.1.2.2 (Zhang et al. 2020), using IQ-TREE v.2.2.0 (Minh et al. 2020) with 1000 standard bootstrap replicates. The GTR+F+I+G4 model was selected by ModelFinder (Kalyaanamoorthy et al. 2017) according to the corrected Akaike Information Criterion (AICc). For ABGD (Puillandre et al. 2012), we used the Jukes-Cantor (JC69) model with a relative gap width X = 1.0, and the rest of parameters set to default (website: https://bioinfo.mnhn.fr/abi/public/abgd/).

**Table 1.** Samples used in this study.

Species	Voucher ID	GenBank accession number	Collection information
**ingroup**			
*A.bimaculata*		OQ736904	Menglun, Yunnan, China
	5002287	PP133869	Menglun, Yunnan, China
	5002288	PP133870	Menglun, Yunnan, China
	5002329, F	PP133873	Menglun, Yunnan, China
	5002286	PP133874	Menglun, Yunnan, China
*A.transversa*		OQ736996	Wuzhishan, Hainan, China
	5002314, F	PP133872	Jianfengling, Hainan, China
*A.bruna*		OQ736905	Puer, Yunnan, China
	5002343, F	PP133875	Jianfengling, Hainan, China
	5002342, F	PP133876	Jianfengling, Hainan, China
*A.xizangensis*		OQ736995	Linzhi, Xizang, China
	5002302, F	PP133871	Linzhi, Xizang, China
*A.robusta*		OQ736903	Limushan, Hainan, China
	5002282, F	PP133867	Puer, Yunnan, China
	5002308, F	PP133868	Puer, Yunnan, China
*A.ornata*		KY349665	
*A.australiensis*		MG882127	
*A.redacta* sp. nov.	5002334	PP133862	Honghe, Yunnan, China
*A.unicaudata* sp. nov.	5002289, F	PP133863	Honghe, Yunnan, China
	5002291, F	PP133866	Honghe, Yunnan, China
	5002290	PP133865	Honghe, Yunnan, China
*A.lunulara* sp. nov.	5015272, F	PP133864	Chuxiong, Yunnan, China
*A.bifolium* sp. nov.	5002309	PP133860	Baoshan, Yunnan, China
*A.hemiptera* sp. nov.	5002310	PP133861	Baoshan, Yunnan, China
		OQ736902	Jianfengling, Hainan, China
	5013913	PP133877	Jianfengling, Hainan, China
**outgroup**			
*Margatteaconcava*		MW970256	
*Baltavilis*		KT279743.1	
*Sorineuchoranigra*		KY349516	
*Shelfordinavolubilis*		KY349562	

Note: “F” after voucher number means female sample, without F is male sample.

## ﻿Results

**Figure 1.**
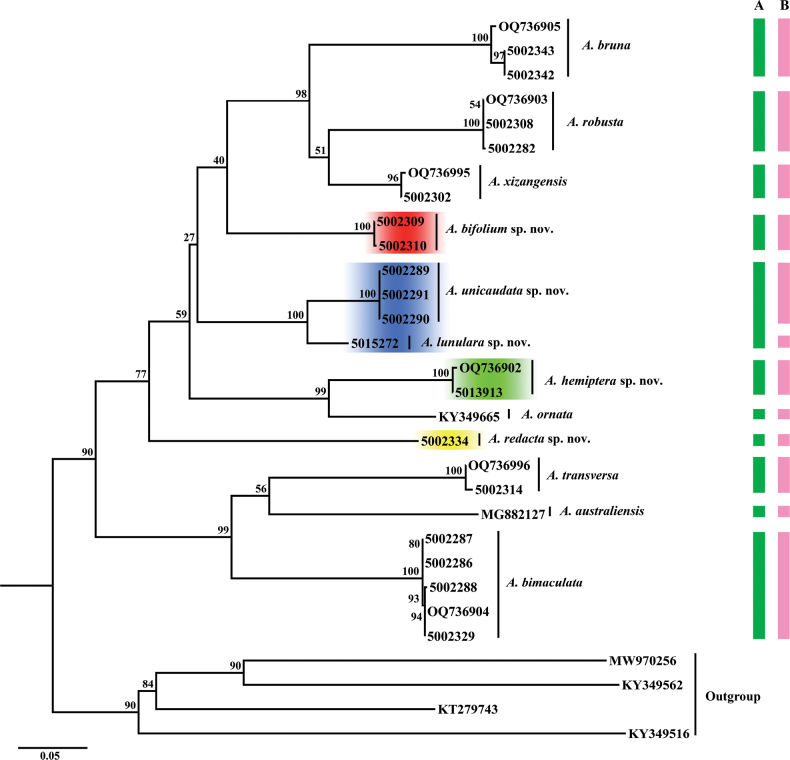
Maximum likelihood (ML) tree based on *COI* sequence. Branch node labels are support values. Colored bars indicate different species delimitation by different methods **A** morphology (green) **B** ABGD results (pink). The colored clades (red, yellow, green and blue highlights) on the tree correspond to four new morphological species.

ML analysis clustered females together with morphologically similar males. We identified 11 morphospecies of *Allacta* on the basis of morphological characters, mainly body color, pronotum pattern, head features, legs, wing venation and male genitalia (Fig. 1A), of which, four new morphological species were identified (four branches with red, yellow, green and blue highlights in Fig. 1). All *Allacta* species were divided into 12 molecular operational taxonomic units (MOTUs) by ABGD analysis as indicated by the pink bar (Fig. 1B). Taxonomic results were identical between morphological delimitation and ABGD except the branch highlighted with blue color, which is a single morphospecies but divided into two MOTUs by ABGD.

### ﻿Taxonomy

#### ﻿ Allacta

Taxon classificationAnimaliaBlattodeaPseudophyllodromiidae

Saussure & Zehntner, 1895

47B6736C-1EB1-54D5-A6C8-CBAE12C72BD7

##### Diagnosis.

The characteristics of the external structure and male genitalia were given in full in Roth (1993b) and He et al. (2019). The following are supplements or adjustments. Tegmina and wings usually fully developed, sometimes reduced (*A.bifolium* Li & Wang, sp. nov. and *A.redacta* Li & Wang, sp. nov.). Subgenital plate usually with two styli, a few with one stylus (*A.unicaudata* Li & Wang, sp. nov.).

##### Remarks.

Species of *Allacta* from China were listed by He et al. (2019); three belong to the *hamifera* species group (*A.alba*, *A.bimaculata* and *A.bruna*) and the remaining four belong to the *polygrapha* species group (*A.ornata*, *A.robusta*, *A.transversa* and *A.xizangensis*) (Roth 1993b; Wang et al. 2014; He et al. 2019). The five new species are placed in the *hamifera* species group by the male interstylar margin being broadly V-shaped (Roth 1993b).

### ﻿Checklist of *Allacta* species from China

***polygrapha* species group**:

*Allactaalba* He, Zheng, Qiu, Che & Wang, 2019: 6. China (Zhejiang).

*Allactabimaculata* Bey-Bienko, 1969: 858. China (Yunnan, Guangxi).

*Allactabruna* He, Zheng, Qiu, Che & Wang, 2019: 4. China (Hainan).

*Allactaxizangensis* Wang, Gui, Che & Wang, 2014: 449. China (Xizang).

***hamifera* species group**:

*Allactaornata* Bey-Bienko, 1969: 859. China (Yunnan, Hainan).

*Allactarobusta* Bey-Bienko, 1969: 860. China (Yunnan).

*Allactatransversa* Bey-Bienko, 1969: 859. China (Hainan); Vietnam.

*Allactabifolium* Li & Wang, sp. nov. China (Yunnan).

*Allactalunulara* Li & Wang, sp. nov. China (Yunnan).

*Allactaredacta* Li & Wang, sp. nov. China (Yunnan).

*Allactaunicaudata* Li & Wang, sp. nov. China (Yunnan).

*Allactahemiptera* Li & Wang, sp. nov. China (Hainan).

### ﻿Key to species of *Allacta* from China

**Table:** 

1	Male interstylar margin broadly V-shaped	**2** (***hamifera*-group**)
–	Male interstylar margin without a keel-like ridge	**9** (***polygrapha*-group**)
2	Tegmina and hind wings reduced, not reaching end of abdomen	**3**
–	Tegmina and hind wings fully developed, both extending beyond end of abdomen	**5**
3	hind wings absent	***A.bifolium* sp. nov.**
–	hind wings present	**4**
4	Body broad, disc of pronotum dark brown with a yellowish vertical stripe in the middle	***A.hemiptera* sp. nov.**
–	Body narrow, disc of pronotum dark brown without vertical stripe	***A.redacta* sp. nov.**
5	Head with two dark brown longitudinal stripes reaching from vertex to frons between the antennal sockets, and subgenital plate with dissimilar styli	***A.robusta* Bey-Bienko, 1969**
–	Head with one dark brown longitudinal stripe reaching from vertex to clypeus or not, and subgenital plate with similar styli	**6**
6	Pronotal disk with an inverted triangular yellowish spot in the middle	**7**
–	Pronotal disk without an inverted triangular yellowish spot in the middle	**8**
7	Face with one vertical wide dark brown stripe	***A.ornata* Bey-Bienko, 1969**
–	Face with three narrow horizontal dark brown stripes	***A.transversa* Bey-Bienko, 1969**
8	Female genitalia with third valves asymmetrical and slender rod-shaped	***A.unicaudata* sp. nov.**
–	Female genitalia with third valves symmetrical and broad crescent-shaped	***A.lunulara* sp. nov.**
9	Subgenital plate symmetrical	***A.bruna* He, Zheng, Qiu, Che & Wang, 2019**
–	Subgenital plate asymmetrical	**10**
10	Pronotal disc brown without maculae	***A.bimaculata* Bey-Bienko, 1969**
–	Pronotal disc with maculae	**11**
11	Pronotal disc with trapezoidal symmetrical white maculae	***A.alba* He, Zheng, Qiu, Che & Wang, 2019**
–	Pronotal disc without large trapezoidal shaped white maculae posteriorly	***A.xizangensis* Wang, Gui, Che & Wang, 2014**

#### ﻿ Allacta bifolium

Taxon classificationAnimaliaBlattodeaPseudophyllodromiidae

Li & Wang sp. nov.

588DAE3D-C799-552C-AD29-CA2134DBE11F

https://zoobank.org/1175CE1D-9C23-4CC9-9EE9-28A2677B9FD3

Fig. 2A–M

##### Type material

**(All in SWU). *Holotype***: China • male; Yunnan Prov., Baoshan City, Baihualing; 1253 m; 24 Aug., 2015; Xin-Ran Li, Zhi-Wei Qiu leg. ***Paratypes***: China • 3 males; same data as holotype; 1 male, Yuxi City, Xinping County, Mount Ailao, 1933 m, 12 May, 2016, Lu Qiu, Zhi-Wei Qiu leg.

##### Diagnosis.

This species can be easily distinguished from its congeners by the small leaf-shaped tegmina, the absence of hind wings as well as the right side of the right stylus with a long, finger-like protrusion.

##### Measurements

**(mm).** Male, pronotum length × width: 3.1–3.2 × 4.9–5.2, tegmina length: 1.9–2.3, overall length: 11.2–12.5.

##### Description.

**Figure 2.**
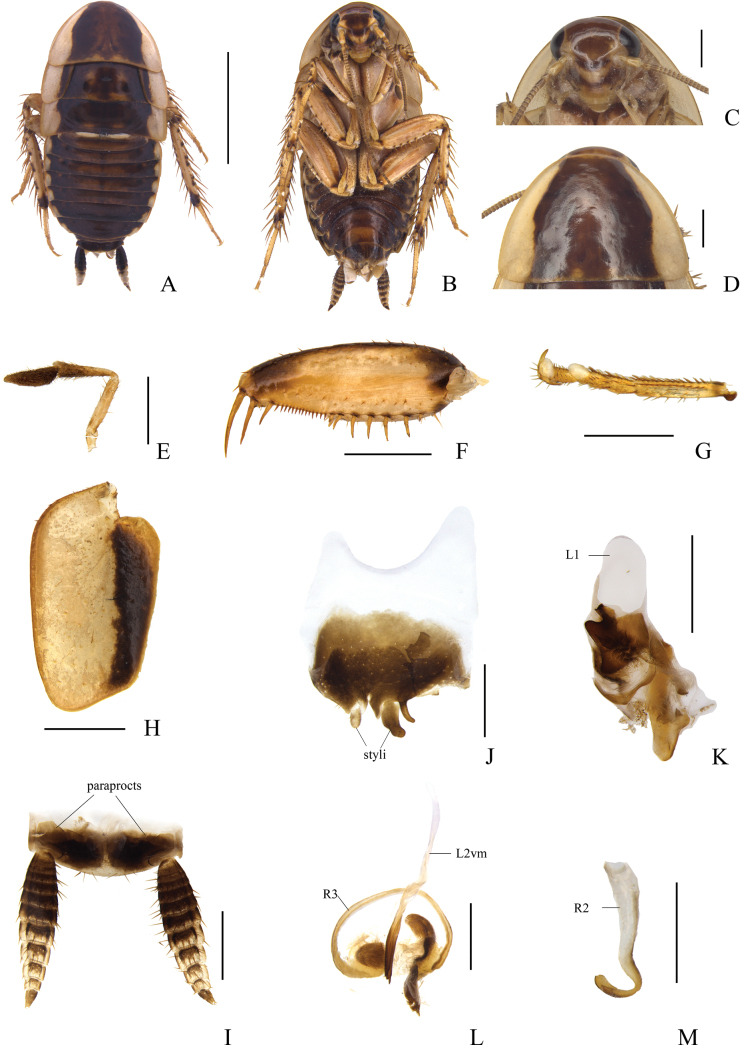
*Allactabifolium* sp. nov., male holotype **A** dorsal view **B** ventral view **C** head, ventral view **D** pronotum, dorsal view **E** maxillary palpi, ventral view **F** front femur, ventral view **G** tarsus and tarsal claws of front leg **H** tegmen, dorsal view **I** supra-anal plate and paraprocts, ventral view **J** subgenital plate, dorsal view **K** left phallomere, dorsal view **L** median phallomere, dorsal view **M** hook-like phallomere, dorsal view. Scale bars: 5 mm (**A, B**); 1 mm (**C–M**).

**Male.** Body dark brown (Fig. 2A, B). Face brown with a yellowish-brown transverse crescent band below antennal sockets (Fig. 2C). Lateral portions of thorax yellowish brown, including pronotum, mesonotum and metanotum as well as most of the tegmina (Fig. 2A). Tibiae yellowish brown with base dark brown. Cerci dorsally with basal half blackish brown, terminal parts yellowish brown; each segment ventrally with basal half dark brown and apical half light brown.

Vertex with interocular space greater than the distance between antennal sockets. The third, and fourth maxillary palpi of approximately the same length, slightly longer than the fifth (Fig. 2E). Pronotum subparabolic with hind margin nearly straight. Tegmina greatly reduced (Fig. 2H), slightly surpass mesonotum, without veins. Hind wings absent. Anteroventral margin of front femur Type B_3_ (Fig. 2F). Pulvillus only present on the fourth tarsomere (Fig. 2G). Tarsal claws symmetrical and unspecialized, arolia present.

***Male abdomen and genitalia*.** Abdominal terga unspecialized. Supra-anal plate short, symmetrical, and hind margin arc-shaped. Paraprocts simple and plate-like (Fig. 2I). Subgenital plate asymmetrical with two styli arising in two concavities of hind margin. The right stylus longer than the left; the right cylindrical with a finger-like projection on right side; the left stylus nearly elliptical (Fig. 2J). The interstylar margin broadly V-shaped. Left phallomere complex (Fig. 2F). Median phallomere (L2vm) stem slender rod-like, slightly curved, apex blunt round with several small spines, base sharp with a large spine subsidiary sclerite; median phallomere subsidiary sclerite (R3) C-shaped clavate (Fig. 2L). Hooked phallomere (R2) on the right of subgenital plate, with pre-apical incision (Fig. 2M).

##### Etymology.

The Latin words *bi*- means pair, double, and *folium* means leaf, referring to the tegmina being degenerated into small leaf-like structures and hind wings absent.

##### Distribution.

China (Yunnan).

#### ﻿ Allacta redacta

Taxon classificationAnimaliaBlattodeaPseudophyllodromiidae

Li & Wang sp. nov.

99DE8FDF-FF5B-5E3E-A3FC-D4444267BC62

https://zoobank.org/43882093-F05A-47B2-8072-E08C323B0FEB

Fig. 3A–M

##### Type material.

***Holotype***: China • male (SWU); Yunnan Prov., Pingbian County, Mount Dawei; 1496 m; 15 May, 2016; Lu Qiu, Zhi-Wei Qiu leg.

##### Diagnosis.

This species can be easily distinguished from its congeners by the wings being reduced and the pronotal disk with a brownish mushroom-shape marking.

##### Measurements

**(mm).** Male, pronotum length × width: 3.3 × 4.5, tegmina length: 4.5, overall length: 13.5.

##### Description.

**Figure 3.**
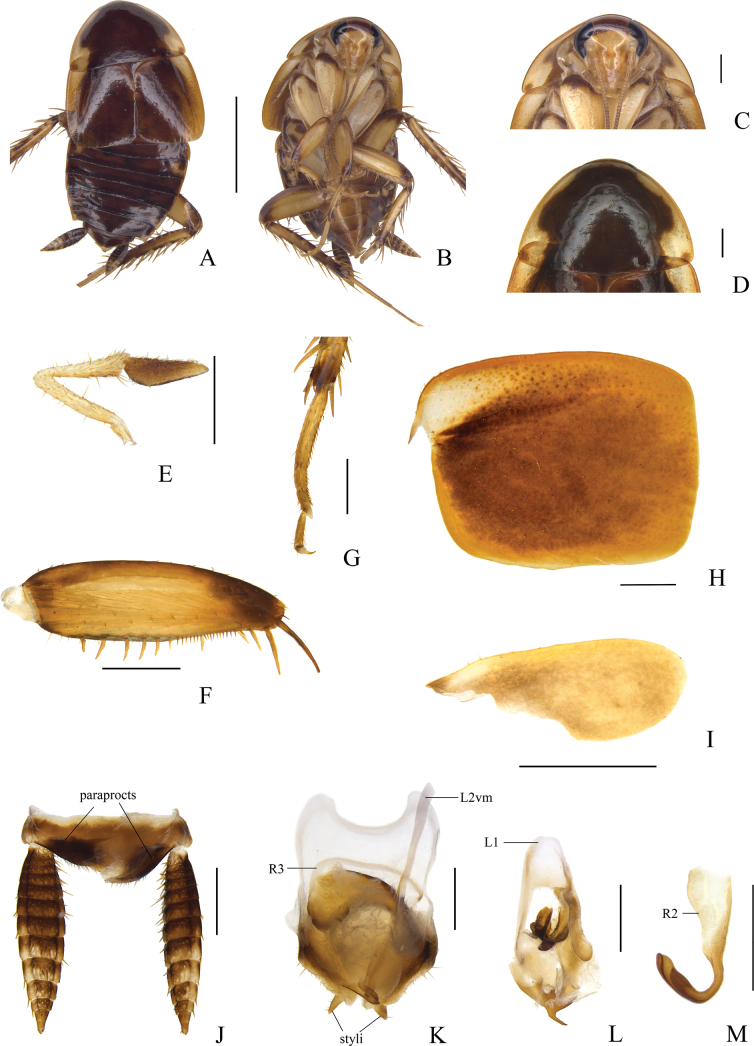
*Allactaredacta* sp. nov., male holotype **A** dorsal view **B** ventral view **C** head, ventral view **D** pronotum, dorsal view **E** maxillary palpi, ventral view **F** front femur, ventral view **G** tarsus and tarsal claws of front leg **H** tegmen, dorsal view **I** hind wing, dorsal view **J** supra-anal plate and paraprocts, ventral view **K** subgenital plate and median phallomere, dorsal view **L** left phallomere, dorsal view **M** hook-like phallomere, dorsal view. Scale bars: 5 mm (**A, B**); 1 mm (**C–M**).

**Male.** Body medium-sized, yellowish brown (Fig. 3A, B). Face yellowish brown with a large brown crescent band; antennae brownish yellow, darkening apically; the fifth maxillary palpus brown, the rest brownish yellow (Fig. 3C). Pronotum dark brown, lateral borders and posterolateral corners of pronotum pale yellowish brown (Fig. 3D). Tegmina yellowish brown. Abdomen terga reddish brown, lateral border light brown; sterna brownish yellow. Subgenital plate with posterior half brown (Fig. 3K). Cerci black in basal half of dorsal surface, and yellowish brown ventrally. Legs brownish yellow, coxae darker, tibiae yellowish with spines attachment area brown (Fig. 3J).

Vertex with interocular space greater than distance between antennal sockets. Pronotum subparabolic with hind margins nearly straight (Fig. 3D). The third and fourth maxillary palpi of approximately same length, slightly longer than the fifth (Fig. 3E). Tegmina and hind wings reduced, tegmina reach anterior edge of the second abdominal tergite; hind wings reach nearly two-thirds length of metanotum, width approximately one-tenth of tegmina (Fig. 3H, I). Anteroventral margin of front femur Type B_3_ (Fig. 3F). Pulvillus only present on the fourth tarsomere (Fig. 3G). Tarsal claws symmetrical and unspecialized, arolia present.

***Male abdomen and genitalia*.** Abdominal terga unspecialized. Supra-anal plate short, nearly triangular, symmetrical, with hind margin blunt round. Paraprocts simple and plate-like, with scattered setae on distal margin (Fig. 3J). Subgenital plate slightly asymmetrical, styli nearly elliptical, arising from the posterior margin concavities, right stylus slightly larger than the left, interstylar margin with broadly V-shaped notch (Fig. 3K). Left phallomere complex (Fig. 3L). Median phallomere (L2vm) stem slender, rod-like, apex blunt round with several small spines, with fine spines and bifurcation at three-quarters from base; median phallomere subsidiary sclerite (R3) C-shaped (Fig. 3K). Hooked phallomere (R2) on the right of subgenital plate, with V-shaped incision (Fig. 3M).

##### Etymology.

The specific name *redacta* derived from Latin, refers to both the tegmina and hind wings being reduced, which do not reach half the length of the normal wings of its congeners.

##### Distribution.

China (Yunnan).

#### ﻿ Allacta unicaudata

Taxon classificationAnimaliaBlattodeaPseudophyllodromiidae

Li & Wang sp. nov.

10C30430-4C81-5253-B0AA-2E6CCEF8A50A

https://zoobank.org/6DFBFA71-EF72-470D-A851-F135D668408D

Fig. 4A–Q

##### Type material

**(All in SWU). *Holotype***: China • male; Yunnan Prov., Pingbian County, Mount Dawei; 1496 m; 15 May, 2016; Lu Qiu, Zhi-Wei Qiu leg. ***Paratypes***: China • 1 male and 1 female, same data as holotype.

##### Diagnosis.

This species can be easily distinguished from all congeners by the absence of the left stylus in males, except for *Allactalunulara* sp. nov., of which males are unknown (see below for females). This species shares a similar appearance with *A.lunulara* sp. nov., but it can be differentiated from the latter mainly by the following characters of the female genitalia: 1) third valves asymmetrical and slender rod-shaped, while symmetrical and broad crescent-shaped in *A.lunulara*; 2) posterior half of basivalvula narrower than the basal half, while basivalvula oval-shaped in *A.lunulara*; 3) spermatheca plate rounded with a sharp protrusion in the middle of the spermatheca plate, while front margin of spermatheca plate truncated in *A.lunulara*; and 4) laterosternal shelf asymmetrical, narrow, long and slightly curved, while symmetrical, broad and triangular in *A.lunulara*.

##### Measurements

**(mm).** Male, pronotum length × width: 3.7–4.0 × 5.5–5.9, tegmina length: 10.9–12.6, overall length: 14.9–16.0; female, pronotum length × width: 3.7–4.4 × 4.6–5.7, tegmina length: 9.2–11.4, overall length: 11.5–14.7.

##### Description.

**Figure 4.**
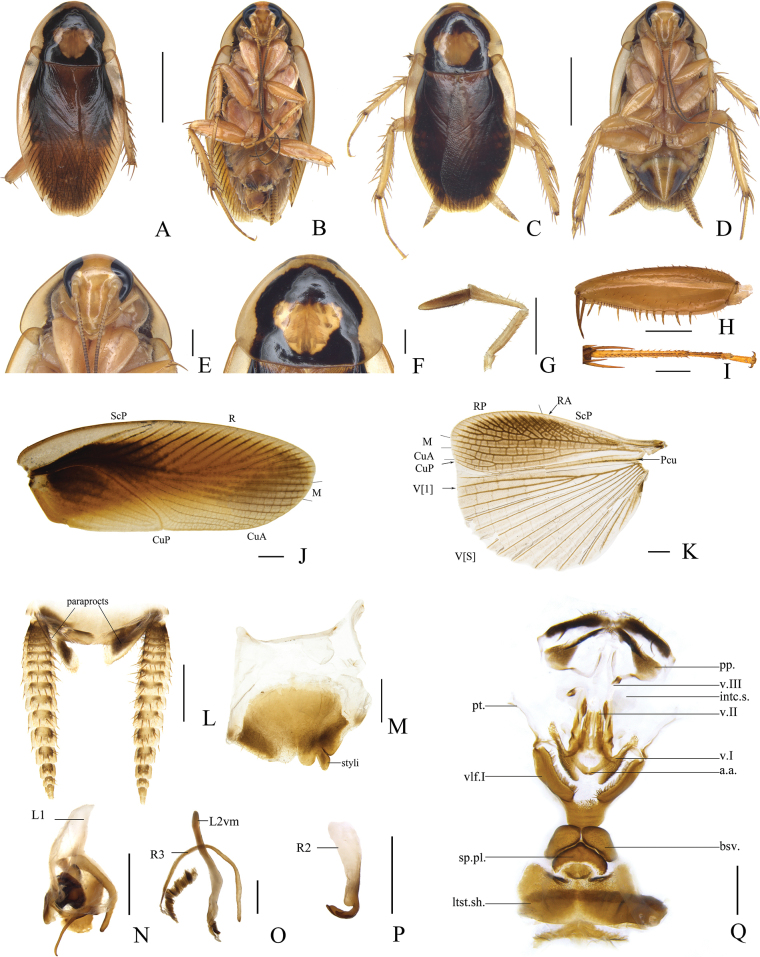
*Allactaunicaudata* sp. nov., male holotype **A** dorsal view **B** ventral view **C** paratype (female), dorsal view **D** paratype (female), ventral view **E** head, ventral view **F** pronotum, dorsal view **G** maxillary palpi, ventral view **H** front femur, ventral view **I** tarsus and tarsal claws of front leg **J** tegmen, dorsal view **K** hind wing, dorsal view **L** supra-anal plate and paraprocts, ventral view **M** subgenital plate, dorsal view **N** left phallomere, dorsal view **O** median phallomere, dorsal view **P** hook-like phallomere, dorsal view **Q** female subgenital plate, dorsal view. Scale bars: 5 mm (**A–D**); 1 mm (**E–Q**). Abbreviations: **a.a.** anterior arch, **bsv.** basivalvula, **intc.s.** intercalary sclerite, **ltst.sh.** laterosternal shelf, **pp.** paraprocts, **pt.** paratergites, **sp.pl.** spermathecal plate, **vlf.I** first valvifer, **v.I** first valves, **v.II** second valves, **v.III** third valves. For vein abbreviations see Material and methods.

**Male.** Body yellowish brown (Fig. 4A–D). Head yellow with ocelli white, frons with a yellowish-brown longitudinal stripe (Fig. 4E). The third and fourth maxillary palpi dark yellow, and the fifth maxillary palpus brown (Fig. 4G). Antennae yellowish brown, darkening apically. Lateral borders and front margin of pronotum translucent yellowish, a dark yellow inverted triangular pattern in the middle (Fig. 4F). Tegmina yellowish orange, clearly uneven in color with radial field, mediocubital field, and anal field darkening basally. Hind wings pale brown (Fig. 4J, K). Legs yellowish brown. Subgenital plate with posterior half grayish yellow (Fig. 4M). Cerci yellowish brown, with basal segment darker (Fig. 4L).

Vertex with interocular space narrower than distance between antennal sockets. The third and fourth maxillary palpi slightly longer than the fifth (Fig. 4G). Pronotum subparabolic with hind margins truncated (Fig. 4F). Tegmina and hind wings fully developed, both extending beyond the end of abdomen. Tegmina slender, with M and CuA longitudinal, CuA with four branches (Fig. 4J). M of hind wings with two branches, CuA curved with three complete branches (Fig. 4K). Anteroventral margin of front femur Type B_3_ (Fig. 4H). Pulvillus only present on the fourth tarsomere. Tarsal claws symmetrical and unspecialized, arolium present.

***Male abdomen and genitalia*.** Abdominal terga unspecialized. Supra-anal plate short, symmetrical, with hind margin slightly concave. Paraprocts simple and plate-like (Fig. 4L). Subgenital plate asymmetrical with a V-shaped notch at the interstylar margin, without left stylus, right stylus cylindrical arising in a concavity of the hind margin near right posterolateral corner (Fig. 4M). Left phallomere complex (Fig. 4N). Median phallomere (L2vm) stem slender, rod-like, slightly curved, apex bluntly round with a small spine; median phallomere subsidiary sclerite (R3) C-shaped, apex sharp with a brush-like structure (Fig. 4O). Hooked phallomere (R2) on the right of subgenital plate, with pre-apical incision (Fig. 4P).

***Female genitalia*.** Supra-anal plate nearly symmetrical. Paraprocts broad. Intercalary sclerite irregular plate-shaped and translucent. First valves robust, with inward protrusions. Second valves small. Third valves asymmetrical, slender rod-shaped, and the left branch obviously curved outward. The anterior margin of anterior arch slightly sclerotized. First valvifer long and narrow plate-like with setae on the inside. Basivalvula spindle-shaped, wide in the middle and tapers at both ends. Laterosternal shelf asymmetrical, narrow, long and slightly curved. Front margin of spermathecal plate rounded with a sharp protrusion in the middle. The spermatheca lobe forked, the end of one spermatheca branch enlarged, and the other long and tubular (Fig. 4R, S).

##### Remarks.

This species resembles *A.alba*, but it can be differentiated from the latter by the following characters: 1) pronotal disc with an inverted triangular yellowish spot, while with subtrapezoidal symmetrical white markings in *A.alba*; 2) tegmina and hind wings slightly extending beyond the end of the abdomen, while extending far beyond the end of the abdomen in *A.alba*; and 3) subgenital plate without left stylus, while with two styli in *A.alba*. This species is placed in the *hamifera* species group by having the male interstylar margin broadly V-shaped.

##### Etymology.

The Latin words *uni*- meaning one, single, and *caudata* meaning tailed, referring to subgenital plate with only one stylus.

##### Distribution.

China (Yunnan).

#### ﻿ Allacta lunulara

Taxon classificationAnimaliaBlattodeaPseudophyllodromiidae

Li & Wang sp. nov.

36E84531-88CD-5589-987B-697760A9A8BA

https://zoobank.org/B362287A-A0F7-4723-9E99-1EE39F9CE78A

Fig. 5A–L

##### Type material

**(All in SWU). *Holotype***: China • female; Yunnan Prov., Chuxiong City, Mount Zixi; 2239 m; 31 Jul., 2022; Lin Guo, Wei Han leg. ***Paratype***: China • 1 female, same data as holotype.

##### Diagnosis.

This species resembles *A.unicaudata*, but it can be differentiated from *A.unicaudata* mainly by the symmetrical and crescent-shaped third valves.

##### Measurements

**(mm).** Female, pronotum length × width: 3.3–3.4 × 4.8–5.2, tegmina length: 8.5–9.0, overall length: 11.6–12.2.

##### Description.

**Figure 5.**
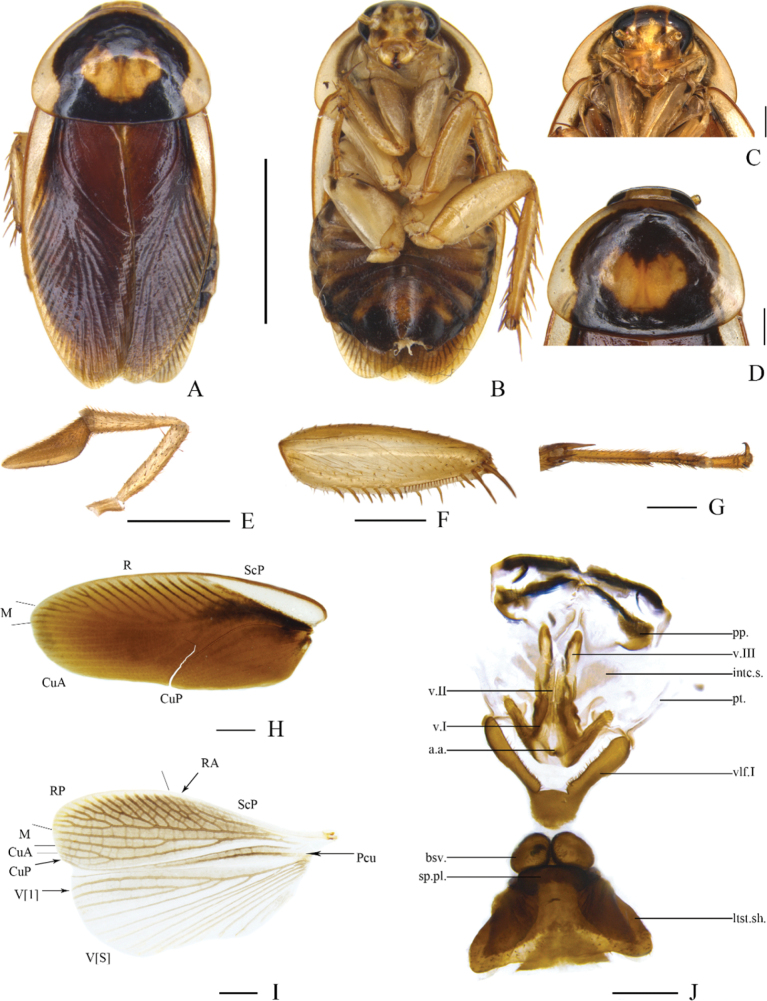
*Allactalunulara* sp. nov., female holotype **A** dorsal view **B** ventral view **C** head, ventral view **D** pronotum, dorsal view **E** maxillary palpi, ventral view **F** front femur, ventral view **G** tarsus and tarsal claws of front leg **H** tegmen, dorsal view **I** hind wing, dorsal view **J** subgenital plate, dorsal view **K** supra-anal plate, dorsal view **L** supra-anal plate, ventral view. Scale bars: 5 mm (**A, B**); 1 mm (**C–L**). Abbreviations: **a.a.** anterior arch, **bsv.** basivalvula,**intc.s.** intercalary sclerite, **ltst.sh.** laterosternal shelf, **pp.** paraprocts, **pt.** paratergites, **sp.pl.** spermathecal plate, **vlf.I** first valvifer, **v.I** first valves, **v.II** second valves, **v.III** third valves. For vein abbreviations see Materials and methods.

**Female.** Body yellowish brown (Fig. 5A, B). Head yellow with ocelli white; stripe between the eyes dark brown. Maxillary palpi light brown. Antennae yellowish brown (Fig. 5C). Lateral borders and front margin of pronotum translucent yellowish; an inverted triangular yellowish spot in the middle (Fig. 5D). Tegmina yellowish brown, and hind wings light brown (Fig. 5H, I). Legs yellowish brown.

Vertex with interocular space narrower than distance between antennal sockets (Fig. 5C). The third and fourth maxillary palpi of approximately same length, slightly longer than the fifth (Fig. 5E). Pronotum subparabolic with hind margins truncated (Fig. 5D). Tegmina and hind wings fully developed, both extending beyond the end of abdomen. Tegmina slender, with M and CuA longitudinal, CuA with four branches. M of hind wings with three branches, CuA curved with three complete branches (Fig. 5H, I). Anteroventral margin of front femur Type B_3_ (Fig. 5F). Pulvilli only present on the fourth tarsomere. Tarsal claws symmetrical and unspecialized, arolium present.

***Female genitalia*.** Supra-anal plate nearly symmetrical. Paraprocts broad, not extending to the posterior margin of supra-anal plate. Intercalary sclerite irregular plate-shaped and translucent. First valves robust, with inward protrusions. Second valves small. Third valves symmetrical and broad crescent-shaped. The anterior margin of anterior arch slightly sclerotized. First valvifer long and narrow plate-like with setae on the inside. Basivalvula oval-shaped. Laterosternal shelf symmetrical, broad and triangle. Front margin of spermathecal plate truncated. The spermatheca lobe forked, the end of one spermatheca branch enlarged, and the other long and tubular (Fig. 5K, L).

##### Remarks.

This species was found to be a cryptic species, very similar to *A.unicaudata* Li & Wang, sp. nov. but it can be differentiated from *A.unicaudata* by the female genitalia characters aforementioned. In this study, after comparing the female genitalia in five *Allacta* species, it is confirmed that the variation in female genitalia can be applied to identify the species of *Allacta*. The *COI* divergence between them (6.6%) is significantly larger than the usual intraspecies distance in *Allacta* (Suppl. material 1). This species is placed in the *hamifera* species group by having a dark pronotum.

##### Etymology.

The specific epithet is derived from the Latin word *lunulara* which means moon-like or relating to a crescent shape, referring to its crescent-shaped third valves.

##### Distribution.

China (Yunnan).

#### ﻿ Allacta hemiptera

Taxon classificationAnimaliaBlattodeaPseudophyllodromiidae

Li & Wang sp. nov.

77FE43E7-8DD5-5D55-9256-C2E2DBD53B0F

https://zoobank.org/3F418EF5-5850-420B-AFCE-CE43A91514FF

Fig. 6A–O

Temnopteryx hainanensis Liu et al., 2017: 179 (*nomen nudum*); Qin and Liu 2019: 175.
Allacta hainanensis : He et al. 2019: 8.

##### Type material.

***Holotype***: China • male (SWU); Hainan Prov., Ledong County, Mount Jianfeng; 997 m; 16 Apr., 2015; Lu Qiu leg. ***Paratypes***: China • 1 male (SEM); Hainan Prov., Changjiang County, Mount Bawang; 1495 m; 22 Sep., 2011; Xian-Wei Liu leg • 1 male (SWU); Hainan Prov., Ledong County, Mount Jianfeng; 1050 m; 6 Jul., 2007; Wei-Wei Zhang leg • 1 female (SWU); Hainan Prov., Ledong County, Mount Jianfeng; 997 m; 16 Apr., 2015; Lu Qiu leg • 3 males & 5 females (SWU); Hainan Prov., Qiongzhong County, Limushan Stone Forest; 585 m; 12 Jul., 2023; Wen-Bo Deng leg • 3 females (SWU); Hainan Prov., Qiongzhong County, Quling Valley; 662 m; 11 Jul., 2023; Yi-Shu Wang leg.

##### Diagnosis.

This species resembles *A.redacta*, but it can be differentiated from *A.redacta* mainly by the pronotal disk with a nib-shaped yellowish spot.

##### Measurements

**(mm).** Male, pronotum length × width: 4.3–4.6 × 6.9–7.4, tegmina length: 5.5–5.7, overall length: 17.0–17.2; female, pronotum length × width: 4.0–4.4 × 6.5–7.4, tegmina length: 5.3–5.8, overall length: 16.9–17.2.

##### Description.

**Figure 6.**
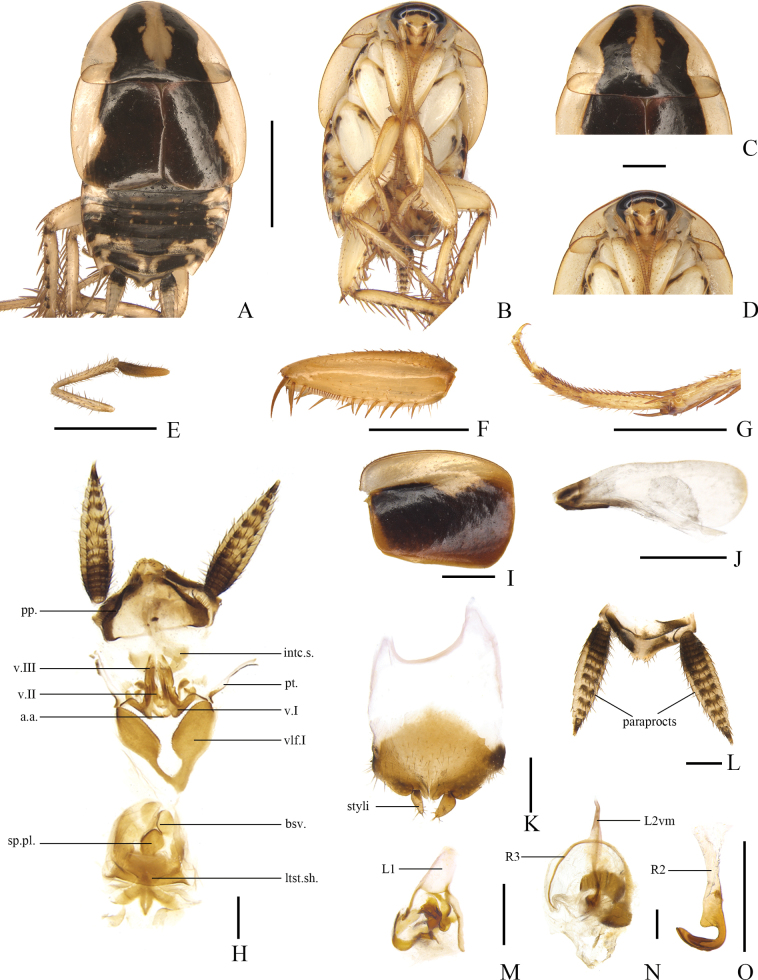
*Allactahemiptera* sp. nov., male holotype **A** dorsal view **B** ventral view **C** pronotum, dorsal view **D** head, ventral view **E** maxillary palpi, ventral view **F** front femur, ventral view **G** tarsus and tarsal claws of front leg **H** female subgenital plate, dorsal view **I** tegmen, dorsal view **J** hind wing, dorsal view **K** subgenital plate, dorsal view **L** supra-anal plate and paraprocts, ventral view **M** left phallomere, dorsal view **N** median phallomere, dorsal view **O** hook-like phallomere, dorsal view. Scale bars: 5 mm (**A, B**); 2 mm (**C–I**); 1 mm (**J–O**). Abbreviations: **a.a.** anterior arch, **bsv.** basivalvula, **intc.s.** intercalary sclerite, **ltst.sh.** laterosternal shelf, **pp.** paraprocts, **pt.** paratergites, **sp.pl.** spermathecal plate, **vlf.I** first valvifer, **v.I** first valves, **v.II** second valves, **v.III** third valves.

**Male.** Body dark brown (Fig. 6A, B). Face brown with dark brown stipples and spots in the middle (Fig. 6D). Antennae brown. The fifth maxillary palpi brown, the rest yellowish brown. Lateral borders and front margin of pronotum translucent yellowish; a nib-shaped yellowish spot in the middle (Fig. 6C). Tegmina brown, lateral borders translucent (Fig. 6I). Legs yellowish brown. Cerci yellowish brown, with basal dark brown (Fig. 6L).

Vertex with interocular space obviously narrower than distance between antennal sockets (Fig. 6D). The third and fourth maxillary palpi of approximately same length, slightly longer than the fifth (Fig. 6E). Pronotum nearly triangle with front margins blunt round and hind margins truncated (Fig. 6C). Tegmina and hind wings reduced. Tegmina nearly quadrilateral, veins not obvious. Hind wings small, thin and transparent, about half the length of tegmina (Fig. 6I, J). Anteroventral margin of front femur Type B_3_ (Fig. 6F). Pulvilli only present on the fourth tarsomere. Tarsal claws symmetrical and unspecialized, arolium present (Fig. 6G).

***Male abdomen and genitalia*.** Abdominal terga unspecialized. Supra-anal plate short, nearly triangular, symmetrical, with incision at the middle of hind margin. Paraprocts simple and plate-like, with scattered setae on distal margin (Fig. 6L). Subgenital plate symmetrical, lateral margins round and styli nearly cylindrical, arising from the posterior margin concavities, right stylus slightly larger than the left, interstylar margin with W-shaped notch (Fig. 6K). Left phallomere complex (Fig. 6M). Median phallomere (L2vm) stem slender, rod-like, apex sharp, with a crack at quarter from base; median phallomere subsidiary sclerite (R3) C-shaped rod-like (Fig. 6N). Hooked phallomere (R2) on the right of subgenital plate, with V-shaped incision (Fig. 6O).

***Female genitalia*.** Supra-anal plate nearly symmetrical. Paraprocts broad, not extending to the posterior margin of supra-anal plate. Intercalary sclerite irregular plate-shaped and translucent. First valves robust, with inward protrusions. Second valves small. Third valves symmetrical and broad rod-shaped. The anterior margin of anterior arch slightly sclerotized. First valvifer irregular swollen and oval with short setae on the inside. Basivalvula oval-shaped. Laterosternal shelf symmetrical, broad and trapezoid. Front margin of spermathecal plate truncated. The spermatheca lobe forked, the end of one spermatheca branch enlarged, and the other long and tubular (Fig. 6H).

##### Remarks.

According to the International Code of Zoological Nomenclature (Article 16.4.2) (ICZN 1999), *Temnopteryxhainanensis*Liu et al., 2017 is invalid. He et al. (2019) did not realize that and moved *Temnopteryxhainanensis* to the genus *Allacta*. We here describe it as a new species based on the new material and the type specimens of *Temnopteryxhainanensis*Liu et al., 2017.

##### Etymology.

The Latin terms *hemi*- means half, *ptera* means wing, and *hemiptera* means that the tegmina is half the normal wing length.

##### Distribution.

China (Hainan).

## ﻿Discussion

**Figure 7.**
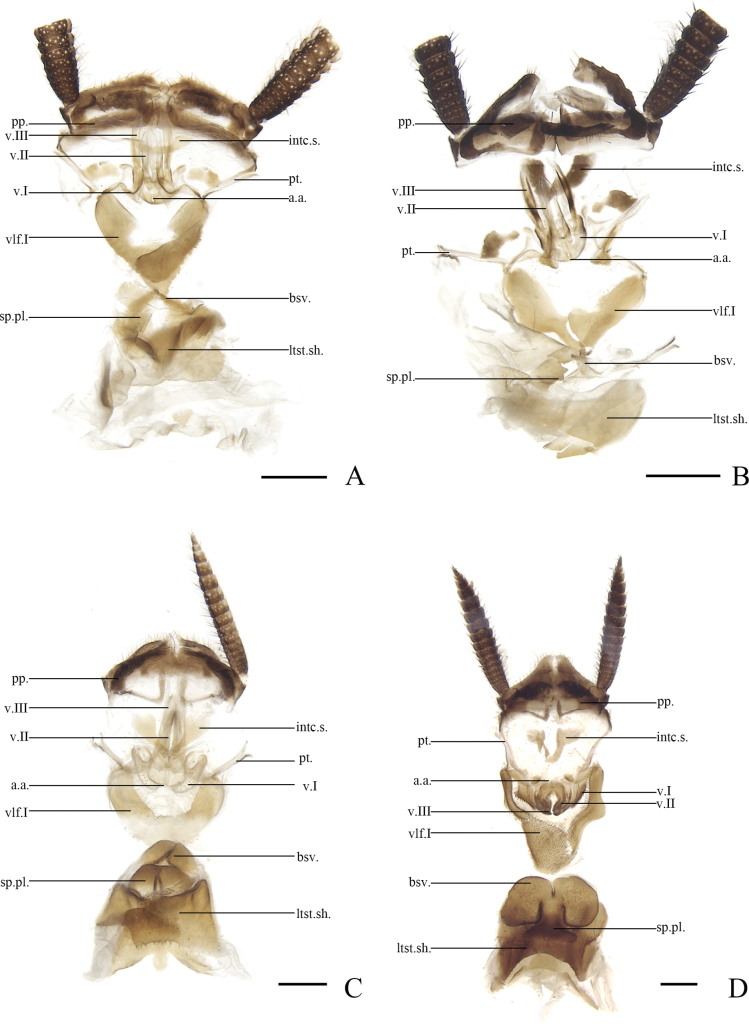
**A***Allactatransversa* Bey-Bienko, 1969. Female **B***Allactabimaculata* Bey-Bienko, 1969. Female **C***Allactarobusta* Bey-Bienko, 1969. Female **D***Allactabruna* He, Zheng, Qiu, Che & Wang, 2019. Female **A–D** supra-anal plate, dorsal view. Scale bars: 1 mm. Abbreviations: **a.a.** anterior arch, **bsv.** basivalvula, **intc.s.** intercalary sclerite, **ltst.sh.** laterosternal shelf, **pp.** paraprocts, **pt.** paratergites, **sp.pl.** spermathecal plate, **vlf.I** first valvifer, **v.I** first valves, **v.II** second valves, **v.III** third valves.

External characteristics and male genitalia have been traditionally used to define species of *Allacta* (Roth 1993b; Wang et al. 2014; He et al. 2019), but identifying species relied too much on male characteristics. For example, the male genitalia of *A.lunulara* Li & Wang, sp. nov. was not available and therefore could not be used to determine whether it was a new species in this study. As such, we tried to look for morphological divergence in female morphology. We compared the female genitalia of four known species and *A.hemiptera* sp. nov., and found that there were significant differences mainly in valvifer, first valvifer, basivalvula and laterosternal shelf (Figs 6, 7), indicating that female genitalia could be used for identification in *Allacta*.

The clade highlighted in blue (Fig. 1) was divided into two MOTUs. We carefully compared the female external genitalia of these two MOTUs and found evidence that they differed in the third valves, basivalvula, spermatheca plate and laterosternal shelf. According to the material sampled here, the maximum intraspecific genetic distances of *Allacta* for *COI* was 1.1%, but the minimum divergence of these two clades reaches 6.6% (Suppl. material 1). Female genitalia differences combined with this larger genetic distance indicated that specimen 5015272 was not *A.unicaudata* Li & Wang, sp. nov. Therefore, we proposed that it is a new species, *A.lunulara* Li & Wang, sp. nov.

In future research, morphology specific to females should be taken into greater consideration, as it played a key role in the discovery of new species in this study. Sometimes female genitalia can even be used to discover cryptic species, for example, Zhu et al. (2022) distinguished three new cryptic species from *Anaplectaomei* through differences in female genitalia.

In this study, the absence of one stylus in the family Pseudophyllodromiidae was observed for the first time (*A.unicaudata* Li & Wang, sp. nov.), but this is not a unique case in Blattodea (Kumar and Prinis 1978; Roth 1989, 1993a), for example, *Blattellaparilis* Walker, 1868, *Symplocodeseuryloba* Zheng et al., 2015 and *Symplocodesridleyi* Shelford, 1913 in Blattellidae; *Pycnoscelusindicus* Fabricius, 1775 and *Pycnoscelusnigra* Brunner von Wattenwyl, 1865 in Blaberidae. However, the causes and mechanisms for this phenomenon remains to be discovered.

## Supplementary Material

XML Treatment for Allacta

XML Treatment for Allacta bifolium

XML Treatment for Allacta redacta

XML Treatment for Allacta unicaudata

XML Treatment for Allacta lunulara

XML Treatment for Allacta hemiptera

## ﻿Additional information

### Author ORCIDs

Bian-Lun Li https://orcid.org/0009-0009-1773-0900

Yan-Li Che https://orcid.org/0000-0003-3214-9494

Zong-Qing Wang https://orcid.org/0000-0001-9413-1105

### Data availability

All of the data that support the findings of this study are available in the main text or Supplementary Information.

## Supplementary materials

Supplementary material 1 Interspecific and intraspecific genetic distances of *Allacta* Data type xls https://binary.pensoft.net/file/979969This dataset is made available under the Open Database License (http://opendatacommons.org/licenses/odbl/1.0/). The Open Database License (ODbL) is a license agreement intended to allow users to freely share, modify, and use this Dataset while maintaining this same freedom for others, provided that the original source and author(s) are credited.Bian-Lun Li, Peng-Hui Hu, Lin Guo, Yan-Li Che, Zong-Qing Wang
